# *tert*-Butyl 6-oxo-2,7-diaza­spiro[4.4]nonane-2-carboxyl­ate

**DOI:** 10.1107/S160053681105046X

**Published:** 2011-11-30

**Authors:** Jie Yang

**Affiliations:** a Microscale Science Institute , Weifang University, Weifang 261061, People’s Republic of China

## Abstract

In the title mol­ecule, C_12_H_20_N_2_O_3_, both five-membered rings are in envelope conformations. In the crystal, N—H⋯O hydrogen bonds link the mol­ecules into chains along [010].

## Related literature

For applications of substituted pyrrolidines, see: Domagala *et al.* (1993 ▶); Pedder *et al.* (1976 ▶); Blanco & Sardina (1994 ▶); Husinec & Savic (2005 ▶). For standard bond lengths, see: Allen *et al.* (1987 ▶).
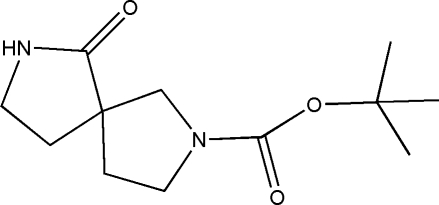


## Experimental

### 

#### Crystal data

C_12_H_20_N_2_O_3_
*M* *_r_* = 240.30
Monoclinic, 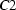

*a* = 10.495 (5) Å
*b* = 6.283 (3) Å
*c* = 19.247 (10) Å
β = 97.029 (8)°
*V* = 1259.7 (11) Å^3^
*Z* = 4
Mo *K*α radiation
μ = 0.09 mm^−1^
*T* = 173 K
0.21 × 0.15 × 0.06 mm

#### Data collection

Rigaku Saturn 724+ diffractometer
Absorption correction: multi-scan (*CrystalClear*; Rigaku, 2007 ▶) *T*_min_ = 0.981, *T*_max_ = 0.995
3265 measured reflections
1557 independent reflections
1452 reflections with *I* > 2σ(*I*)
*R*_int_ = 0.039

#### Refinement

*R*[*F* ^2^ > 2σ(*F* ^2^)] = 0.050
*wR*(*F* ^2^) = 0.105
*S* = 1.09
1557 reflections
157 parameters
1 restraint
H-atom parameters constrained
Δρ_max_ = 0.23 e Å^−3^
Δρ_min_ = −0.18 e Å^−3^

### 

Data collection: *CrystalClear* (Rigaku, 2007 ▶); cell refinement: *CrystalClear*; data reduction: *CrystalClear*; program(s) used to solve structure: *SHELXS97* (Sheldrick, 2008 ▶); program(s) used to refine structure: *SHELXL97* (Sheldrick, 2008 ▶); molecular graphics: *SHELXTL* (Sheldrick, 2008 ▶); software used to prepare material for publication: *SHELXTL*.

## Supplementary Material

Crystal structure: contains datablock(s) global, I. DOI: 10.1107/S160053681105046X/lh5363sup1.cif

Structure factors: contains datablock(s) I. DOI: 10.1107/S160053681105046X/lh5363Isup2.hkl

Additional supplementary materials:  crystallographic information; 3D view; checkCIF report

## Figures and Tables

**Table 1:** Hydrogen-bond geometry (Å, °)

*D*—H⋯*A*	*D*—H	H⋯*A*	*D*⋯*A*	*D*—H⋯*A*
N1—H1⋯O1^i^	0.88	1.97	2.848 (3)	175

Symmetry code: (i) 
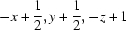
.

**Fig. 1.**
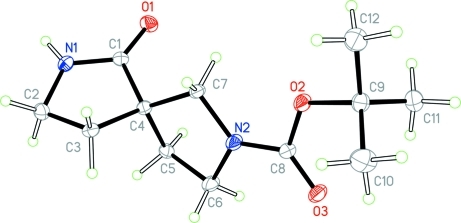
The molecular structure of the title compound with displacement ellipsoids are drawn at the 30% probability level.

## supplementary crystallographic information

### Comment

Depending on the substitution pattern and functionalization, different substituted pyrrolidines have been shown to be effective antibacterials or fungicides agents and glycosidase inhibitors (Domagala  *et al.*, 1993; Pedder *et al.*, 1976; Blanco *et al.*, 1994); Husinec *et al.*, 2005). The crystal structure of the title compound is reportede herein.

In the molecule (Fig. 1), all bond lengths and angles are within normal ranges (Allen *et al.*, 1987). Both five-membered rings are in envelope conformations with C3 and C5 forming the flap. Atoms C6-C8/O2/O3/N2 are essentially planar, with a maximum deviation of 0.0082 (24) Å. In the crystal, N—H···O hydrogen bonds link molecules to form one dimensional chains along [010] (see Table 1).

### Experimental

Tert-butyl 6-oxo-2,7-diazasiro[4.4]nonane-2-carboxylate was synthesized with methyl 1-tert-butyl 3-ethyl 3-(cyanomethyl)pyrrolidine-1,3-dicarboxylate (13.4g) and Raney Ni (3.4g) in methanol under H2(50 Psi) atmosphere at room temperature.

Single crystals of the compound suitable for X-ray measurements were obtained by recrystallization from ethanol at room temperature. In the absence of anomalous dispersion effects the Friedel pairs were merged.

### Refinement

All H atoms were fixed geometrically and allowed to ride on their attached atoms, with C—H distances in the range 0.98–0.99 Å, and with *U*_iso_(H) = 1.2*U*_eq_(C) or *U*_iso_(H) = 1.5*U*_eq_(C_methyl_). The N—H distance is 0.88 Å, with *U*_iso_(H) = 1.2*U*_eq_(N).

### Crystal data

**Table:**

C_12_H_20_N_2_O_3_	*F*(000) = 520
*M**_r_* = 240.30	*D*_x_ = 1.267 Mg m^−^^3^
Monoclinic, *C*2	Mo *K*α radiation, λ = 0.71073 Å
Hall symbol: C 2y	Cell parameters from 2422 reflections
*a* = 10.495 (5) Å	θ = 1.1–27.5°
*b* = 6.283 (3) Å	µ = 0.09 mm^−^^1^
*c* = 19.247 (10) Å	*T* = 173 K
β = 97.029 (8)°	Platelet, colorless
*V* = 1259.7 (11) Å^3^	0.21 × 0.15 × 0.06 mm
*Z* = 4	

### Data collection

**Table:**

Rigaku Saturn 724+ diffractometer	1557 independent reflections
Radiation source: rotating anode	1452 reflections with *I* > 2σ(*I*)
Confocal	*R*_int_ = 0.039
ω scans at fixed χ = 45°	θ_max_ = 27.5°, θ_min_ = 2.1°
Absorption correction: multi-scan (*CrystalClear*; Rigaku, 2007)	*h* = −13→7
*T*_min_ = 0.981, *T*_max_ = 0.995	*k* = −8→8
3265 measured reflections	*l* = −23→25

### Refinement

**Table:**

Refinement on *F*^2^	Primary atom site location: structure-invariant direct methods
Least-squares matrix: full	Secondary atom site location: difference Fourier map
*R*[*F*^2^ > 2σ(*F*^2^)] = 0.050	Hydrogen site location: inferred from neighbouring sites
*wR*(*F*^2^) = 0.105	H-atom parameters constrained
*S* = 1.09	*w* = 1/[σ^2^(*F*_o_^2^) + (0.032*P*)^2^ + 0.9713*P*] where *P* = (*F*_o_^2^ + 2*F*_c_^2^)/3
1557 reflections	(Δ/σ)_max_ < 0.001
157 parameters	Δρ_max_ = 0.23 e Å^−^^3^
1 restraint	Δρ_min_ = −0.18 e Å^−^^3^

### Special details

**Table:**

Geometry. All e.s.d.'s (except the e.s.d. in the dihedral angle between two l.s. planes) are estimated using the full covariance matrix. The cell e.s.d.'s are taken into account individually in the estimation of e.s.d.'s in distances, angles and torsion angles; correlations between e.s.d.'s in cell parameters are only used when they are defined by crystal symmetry. An approximate (isotropic) treatment of cell e.s.d.'s is used for estimating e.s.d.'s involving l.s. planes.
Refinement. Refinement of *F*^2^ against ALL reflections. The weighted *R*-factor *wR* and goodness of fit *S* are based on *F*^2^, conventional *R*-factors *R* are based on *F*, with *F* set to zero for negative *F*^2^. The threshold expression of *F*^2^ > σ(*F*^2^) is used only for calculating *R*-factors(gt) *etc*. and is not relevant to the choice of reflections for refinement. *R*-factors based on *F*^2^ are statistically about twice as large as those based on *F*, and *R*- factors based on ALL data will be even larger.Absolute configuration is unknown, there being no firm chemical evidence for its assignment to hand and it having not been established by anomalous dispersion effects in diffraction measurements on the crystal. An arbitrary choice of enantiomer has been made.

### Fractional atomic coordinates and isotropic or equivalent isotropic displacement parameters (Å^2^)

**Table:**

	*x*	*y*	*z*	*U*_iso_*/*U*_eq_	
O1	0.2715 (2)	0.4600 (3)	0.43850 (10)	0.0313 (5)	
O2	0.33362 (17)	0.2042 (3)	0.18571 (9)	0.0289 (5)	
O3	0.55006 (19)	0.1382 (4)	0.19625 (10)	0.0332 (5)	
N1	0.3406 (2)	0.8081 (4)	0.44345 (11)	0.0255 (5)	
H1	0.3028	0.8474	0.4798	0.031*	
N2	0.4618 (2)	0.3546 (4)	0.27139 (12)	0.0275 (5)	
C1	0.3315 (3)	0.6117 (5)	0.41741 (13)	0.0219 (6)	
C2	0.4181 (3)	0.9527 (5)	0.40704 (14)	0.0279 (6)	
H2B	0.5037	0.9751	0.4339	0.033*	
H2A	0.3750	1.0920	0.3984	0.033*	
C3	0.4286 (3)	0.8347 (5)	0.33812 (13)	0.0234 (6)	
H3B	0.5134	0.8596	0.3220	0.028*	
H3A	0.3605	0.8813	0.3011	0.028*	
C4	0.4118 (3)	0.5993 (5)	0.35651 (13)	0.0209 (5)	
C5	0.5419 (3)	0.4915 (5)	0.38093 (13)	0.0241 (6)	
H5B	0.6051	0.5963	0.4027	0.029*	
H5A	0.5317	0.3766	0.4150	0.029*	
C6	0.5835 (3)	0.4018 (5)	0.31360 (15)	0.0303 (7)	
H6B	0.6336	0.5078	0.2902	0.036*	
H6A	0.6357	0.2714	0.3230	0.036*	
C7	0.3515 (2)	0.4573 (5)	0.29695 (13)	0.0233 (6)	
H7B	0.2936	0.3505	0.3143	0.028*	
H7A	0.3024	0.5428	0.2596	0.028*	
C8	0.4568 (2)	0.2247 (5)	0.21527 (13)	0.0244 (6)	
C9	0.3028 (3)	0.0912 (5)	0.11865 (14)	0.0290 (7)	
C10	0.3776 (3)	0.1875 (7)	0.06389 (15)	0.0461 (9)	
H10A	0.3444	0.1321	0.0176	0.069*	
H10C	0.4686	0.1501	0.0745	0.069*	
H10B	0.3683	0.3427	0.0641	0.069*	
C11	0.3283 (3)	−0.1464 (6)	0.12960 (17)	0.0381 (8)	
H11B	0.2717	−0.2031	0.1620	0.057*	
H11C	0.4180	−0.1680	0.1492	0.057*	
H11A	0.3117	−0.2205	0.0846	0.057*	
C12	0.1607 (3)	0.1350 (7)	0.10196 (17)	0.0419 (8)	
H12A	0.1281	0.0648	0.0579	0.063*	
H12C	0.1466	0.2888	0.0974	0.063*	
H12B	0.1154	0.0799	0.1398	0.063*	

### Atomic displacement parameters (Å^2^)

**Table:**

	*U*^11^	*U*^22^	*U*^33^	*U*^12^	*U*^13^	*U*^23^
O1	0.0348 (12)	0.0317 (11)	0.0293 (10)	−0.0073 (10)	0.0115 (8)	0.0027 (10)
O2	0.0228 (10)	0.0378 (11)	0.0253 (9)	0.0021 (10)	0.0002 (7)	−0.0106 (10)
O3	0.0261 (11)	0.0410 (13)	0.0335 (10)	0.0050 (10)	0.0074 (8)	−0.0098 (10)
N1	0.0263 (12)	0.0276 (13)	0.0234 (11)	0.0030 (11)	0.0066 (9)	−0.0023 (11)
N2	0.0190 (11)	0.0345 (13)	0.0284 (11)	0.0033 (11)	0.0004 (9)	−0.0091 (11)
C1	0.0200 (13)	0.0256 (13)	0.0200 (11)	−0.0001 (12)	0.0015 (10)	0.0016 (11)
C2	0.0280 (15)	0.0235 (13)	0.0322 (14)	0.0000 (13)	0.0033 (11)	0.0014 (13)
C3	0.0197 (13)	0.0265 (14)	0.0247 (12)	0.0022 (12)	0.0059 (10)	0.0037 (12)
C4	0.0193 (12)	0.0224 (13)	0.0211 (11)	0.0013 (12)	0.0025 (10)	0.0009 (11)
C5	0.0206 (13)	0.0249 (14)	0.0262 (13)	−0.0010 (12)	0.0001 (10)	−0.0014 (12)
C6	0.0183 (13)	0.0383 (18)	0.0336 (14)	0.0040 (13)	0.0001 (11)	−0.0076 (13)
C7	0.0181 (13)	0.0271 (13)	0.0252 (12)	0.0021 (12)	0.0053 (10)	−0.0021 (12)
C8	0.0219 (13)	0.0270 (14)	0.0248 (13)	0.0016 (12)	0.0048 (10)	−0.0003 (12)
C9	0.0309 (15)	0.0351 (16)	0.0212 (13)	−0.0022 (14)	0.0033 (11)	−0.0045 (13)
C10	0.048 (2)	0.062 (3)	0.0283 (15)	−0.009 (2)	0.0058 (14)	0.0031 (17)
C11	0.0388 (18)	0.0359 (17)	0.0394 (17)	−0.0022 (16)	0.0030 (14)	−0.0081 (15)
C12	0.0337 (18)	0.050 (2)	0.0395 (17)	0.0044 (17)	−0.0073 (14)	−0.0070 (17)

### Geometric parameters (Å, °)

**Table:**

O1—C1	1.238 (3)	C5—C6	1.525 (4)
O2—C8	1.353 (3)	C5—H5B	0.9900
O2—C9	1.474 (3)	C5—H5A	0.9900
O3—C8	1.214 (3)	C6—H6B	0.9900
N1—C1	1.331 (4)	C6—H6A	0.9900
N1—C2	1.454 (4)	C7—H7B	0.9900
N1—H1	0.8800	C7—H7A	0.9900
N2—C8	1.350 (3)	C9—C12	1.511 (4)
N2—C6	1.458 (4)	C9—C10	1.516 (4)
N2—C7	1.462 (3)	C9—C11	1.526 (5)
C1—C4	1.527 (4)	C10—H10A	0.9800
C2—C3	1.535 (4)	C10—H10C	0.9800
C2—H2B	0.9900	C10—H10B	0.9800
C2—H2A	0.9900	C11—H11B	0.9800
C3—C4	1.536 (4)	C11—H11C	0.9800
C3—H3B	0.9900	C11—H11A	0.9800
C3—H3A	0.9900	C12—H12A	0.9800
C4—C7	1.527 (4)	C12—H12C	0.9800
C4—C5	1.545 (4)	C12—H12B	0.9800
			
C8—O2—C9	120.7 (2)	N2—C6—H6A	111.1
C1—N1—C2	114.6 (2)	C5—C6—H6A	111.1
C1—N1—H1	122.7	H6B—C6—H6A	109.1
C2—N1—H1	122.7	N2—C7—C4	103.8 (2)
C8—N2—C6	121.0 (2)	N2—C7—H7B	111.0
C8—N2—C7	125.5 (2)	C4—C7—H7B	111.0
C6—N2—C7	113.5 (2)	N2—C7—H7A	111.0
O1—C1—N1	127.3 (3)	C4—C7—H7A	111.0
O1—C1—C4	124.3 (3)	H7B—C7—H7A	109.0
N1—C1—C4	108.4 (2)	O3—C8—N2	123.9 (3)
N1—C2—C3	102.6 (2)	O3—C8—O2	126.5 (3)
N1—C2—H2B	111.2	N2—C8—O2	109.6 (2)
C3—C2—H2B	111.2	O2—C9—C12	101.8 (2)
N1—C2—H2A	111.2	O2—C9—C10	109.8 (3)
C3—C2—H2A	111.2	C12—C9—C10	111.2 (3)
H2B—C2—H2A	109.2	O2—C9—C11	109.5 (2)
C2—C3—C4	104.1 (2)	C12—C9—C11	111.1 (3)
C2—C3—H3B	110.9	C10—C9—C11	112.9 (3)
C4—C3—H3B	110.9	C9—C10—H10A	109.5
C2—C3—H3A	110.9	C9—C10—H10C	109.5
C4—C3—H3A	110.9	H10A—C10—H10C	109.5
H3B—C3—H3A	109.0	C9—C10—H10B	109.5
C1—C4—C7	112.9 (2)	H10A—C10—H10B	109.5
C1—C4—C3	102.6 (2)	H10C—C10—H10B	109.5
C7—C4—C3	116.0 (2)	C9—C11—H11B	109.5
C1—C4—C5	109.8 (2)	C9—C11—H11C	109.5
C7—C4—C5	104.0 (2)	H11B—C11—H11C	109.5
C3—C4—C5	111.7 (2)	C9—C11—H11A	109.5
C6—C5—C4	103.8 (2)	H11B—C11—H11A	109.5
C6—C5—H5B	111.0	H11C—C11—H11A	109.5
C4—C5—H5B	111.0	C9—C12—H12A	109.5
C6—C5—H5A	111.0	C9—C12—H12C	109.5
C4—C5—H5A	111.0	H12A—C12—H12C	109.5
H5B—C5—H5A	109.0	C9—C12—H12B	109.5
N2—C6—C5	103.1 (2)	H12A—C12—H12B	109.5
N2—C6—H6B	111.1	H12C—C12—H12B	109.5
C5—C6—H6B	111.1		
			
C2—N1—C1—O1	−179.7 (3)	C7—N2—C6—C5	15.7 (3)
C2—N1—C1—C4	1.7 (3)	C4—C5—C6—N2	−30.5 (3)
C1—N1—C2—C3	15.7 (3)	C8—N2—C7—C4	−175.1 (3)
N1—C2—C3—C4	−25.8 (3)	C6—N2—C7—C4	5.9 (3)
O1—C1—C4—C7	37.5 (4)	C1—C4—C7—N2	−143.8 (2)
N1—C1—C4—C7	−143.8 (2)	C3—C4—C7—N2	98.2 (3)
O1—C1—C4—C3	163.1 (3)	C5—C4—C7—N2	−24.9 (3)
N1—C1—C4—C3	−18.2 (3)	C6—N2—C8—O3	0.5 (4)
O1—C1—C4—C5	−78.0 (3)	C7—N2—C8—O3	−178.4 (3)
N1—C1—C4—C5	100.7 (3)	C6—N2—C8—O2	179.2 (3)
C2—C3—C4—C1	26.7 (3)	C7—N2—C8—O2	0.3 (4)
C2—C3—C4—C7	150.2 (2)	C9—O2—C8—O3	−8.6 (4)
C2—C3—C4—C5	−90.8 (2)	C9—O2—C8—N2	172.7 (2)
C1—C4—C5—C6	155.7 (2)	C8—O2—C9—C12	−172.4 (3)
C7—C4—C5—C6	34.6 (3)	C8—O2—C9—C10	−54.6 (4)
C3—C4—C5—C6	−91.2 (3)	C8—O2—C9—C11	69.9 (3)
C8—N2—C6—C5	−163.3 (3)		

### Hydrogen-bond geometry (Å, °)

**Table:**

*D*—H···*A*	*D*—H	H···*A*	*D*···*A*	*D*—H···*A*
N1—H1···O1^i^	0.88	1.97	2.848 (3)	175.

Symmetry codes: (i) −*x*+1/2, *y*+1/2, −*z*+1.
